# Transcontinental Phylogeography of the *Daphnia pulex* Species Complex

**DOI:** 10.1371/journal.pone.0046620

**Published:** 2012-10-03

**Authors:** Teresa J. Crease, Angela R. Omilian, Katie S. Costanzo, Derek J. Taylor

**Affiliations:** 1 Department of Integrative Biology, University of Guelph, Guelph, Ontario, Canada; 2 Department of Biological Sciences, The State University of New York at Buffalo, Buffalo, New York, United States of America; 3 Department of Pathology, Roswell Park Cancer Institute, Buffalo, New York, United States of America; 4 Department of Biology, Canisius College, Buffalo, New York, United States of America; George Washington University, United States of America

## Abstract

*Daphnia pulex* is quickly becoming an attractive model species in the field of ecological genomics due to the recent release of its complete genome sequence, a wide variety of new genomic resources, and a rich history of ecological data. Sequences of the mitochondrial *NADH dehydrogenase* subunit 5 and *cytochrome* c *oxidase* subunit 1 genes were used to assess the global phylogeography of this species, and to further elucidate its phylogenetic relationship to other members of the *Daphnia pulex* species complex. Using both newly acquired and previously published data, we analyzed 398 individuals from collections spanning five continents. Eleven strongly supported lineages were found within the *D. pulex* complex, and one lineage in particular, panarctic *D. pulex*, has very little phylogeographical structure and a near worldwide distribution. Mismatch distribution, haplotype network, and population genetic analyses are compatible with a North American origin for this lineage and subsequent spatial expansion in the Late Pleistocene. In addition, our analyses suggest that dispersal between North and South America of this and other species in the *D. pulex* complex has occurred multiple times, and is predominantly from north to south. Our results provide additional support for the evolutionary relationships of the eleven main mitochondrial lineages of the *D. pulex* complex. We found that the well-studied panarctic *D. pulex* is present on every continent except Australia and Antarctica. Despite being geographically very widespread, there is a lack of strong regionalism in the mitochondrial genomes of panarctic *D. pulex* – a pattern that differs from that of most studied cladocerans. Moreover, our analyses suggest recent expansion of the panarctic *D. pulex* lineage, with some continents sharing haplotypes. The hypothesis that hybrid asexuality has contributed to the recent and unusual geographic success of the panarctic *D. pulex* lineage warrants further study.

## Introduction

Scientific understanding of the freshwater crustacean *Daphnia* is unusually deep in ecology and toxicology [1], [2], and with the release of a *Daphnia* genome sequence [3], this organism has now become a promising model system with which to investigate a wide range of biological phenomena [4]–[7]. The *Daphnia* genome sequence was derived from a member of the *Daphnia pulex* species complex, which is common throughout the Holarctic, but is also found in South America and Africa [8], [9]. *Daphnia* typically reproduce by cyclic parthenogenesis, in which production of direct-developing embryos by apomixis alternates with production of diapausing eggs via meiosis and fertilization. However, some lineages in the *D. pulex* species complex also produce their diapausing eggs apomictically (obligate parthenogenesis), and have therefore lost the capacity for sexual reproduction. To date, obligate parthenogenesis has not been identified in any other lineages in the subgenus *Daphnia* [10].

Based on morphology, the *D. pulex* species complex was originally thought to include few species with extremely broad geographic distributions, but studies of mitochondrial DNA (mtDNA) variation have revealed three major groups (pulex, pulicaria, and tenebrosa) and at least 12 named lineages (1 in the pulex group, 9 in the pulicaria group, and 2 in the tenebrosa group) of which all but four are found only on one continent [8]. Even so, the geographic limits of some lineages are unknown because of limited genetic analysis and sampling in many parts of the world [9], [11], [12], [13], [14], [15], [16]. In addition, the origin of the most speciose pulicaria group is controversial. Brooks [17] proposed either a North American or Eurasian origin based on greater abundance on these continents compared to southern hemisphere continents. The absence of sexual lineages outside the Holarctic, and higher levels of genetic diversity in the northern hemisphere also provides evidence for a Holarctic origin [9], [18]. However, Mergeay et al. [13] recently proposed a South American origin for the pulicaria group based on phylogenetic evidence showing that its two most divergent lineages are restricted to South America.

One mtDNA lineage in the pulicaria group, panarctic *D. pulex*, has been reported from temperate and arctic North America, the northeastern Palearctic, and Africa [9], [12], [16]. However, this lineage is unknown from most of the Palearctic and Alaska [15]. Furthermore, the basic phylogeographic structure of the panarctic *D. pulex* lineage is unclear. The formation of sublineages has been attributed to Pleistocene or older vicariance events [12]. Hebert et al. [19] first proposed two separate glacial refugia for panarctic *D. pulex* based on the geographic pattern of breeding system, with obligate parthenogenesis having arisen in this taxon in an eastern North America refugium. Later, three distinct glacial refugia were proposed based on regional phylogenetic lineages and patterns of mtDNA nucleotide diversity in relation to refugial locations [14]. Lynch et al. [20] also found well-supported geographic sublineages in North America, and estimated the age of the oldest obligately parthenogenetic lineage of panarctic *D. pulex* (Quebec and New Brunswick) to be only 172,000 generations (Late Glacial).

In the present study, we employ new mtDNA data from expanded geographic surveys to further examine the phylogeographic history of the *D. pulex* species complex. We aimed to address conflicting hypotheses on the phylogeny of this group by combining data from previous studies with new data from vast geographic regions including those that were not well represented previously. Specifically, we aim to investigate (1) the geographic distribution of the mtDNA lineages within this complex, (2) the origin of the pulicaria group, and (3) the phylogeographic history of panarctic *D. pulex*.

## Results

### Phylogenetic Analyses

**Figure 1:**
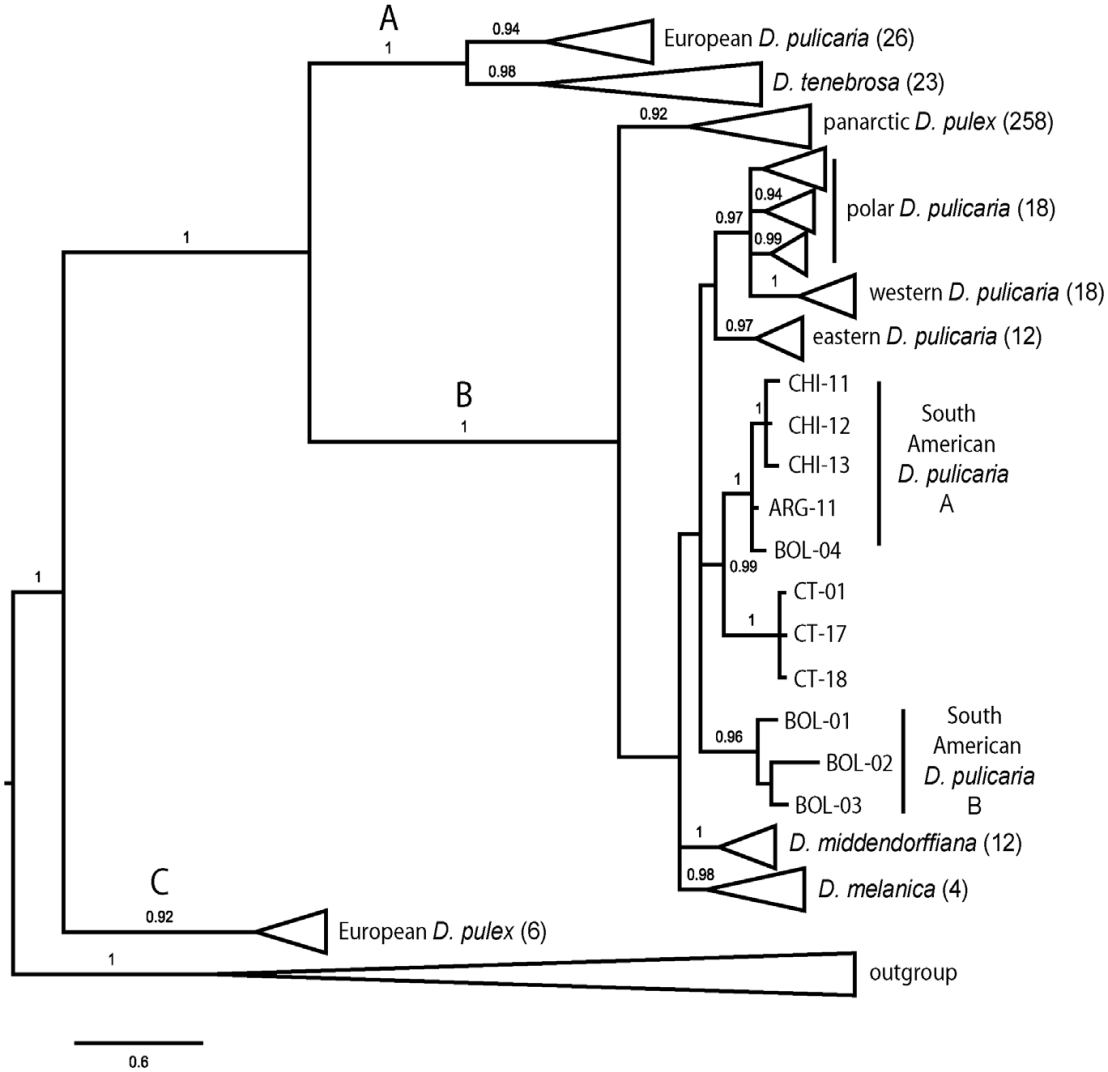
Consensus Bayesian phylogeny of the *Daphnia pulex* species complex based on the mitochondrial ND5 gene. The alignment contains 398 sequences of length 496 nt with 241 polymorphic nt positions of which 204 are phylogenetically informative (excluding the outgroup). Posterior probabilities are indicated on the nodes of the tree and are not shown if less than 0.80. The tree is rooted using an ND5 sequence from *Daphnia obtusa.* Triangles represent clusters that are collapsed to save space and the number of individuals included is shown in parentheses. The large letters indicate the three major groups within the *D. pulex* species complex: A = tenebrosa; B = pulicaria; C = pulex. The two or three-letter code names of some individuals correspond to sampling locations as follows: ARG = Argentina, BOL = Bolivia, CHI = Chile, CT = Connecticut, USA. The expanded version of this tree showing all individuals is available in Figure S1.

Bayesian phylogenetic analysis of a dataset containing 398 partial sequences of the mitochondrial *NADH dehydrogenase* subunit 5 (ND5) gene (Figure 1) reveals strong support for the three major groups within the *D. pulex* species complex [12]: pulex (posterior probability [PP] = 0.92), pulicaria (PP = 1), and tenebrosa (PP = 1). In addition, previously described lineages [12], [13] within these major groups are identified with high posterior probability values, although relationships among lineages are weakly supported. Synonymous site diversity ranges from 0.00 to 0.04 in all lineages except *D. tenebrosa*, which has extremely high diversity at 0.106 (Table 1).

**Table 1:** Polymorphism statistics for ND5 and COX1 (panarctic *D. pulex*) in lineages of the *Daphnia pulex* species complex.

Lineage	N^1^	K^2^	H^3^	S^4^	π_s_ 5	π_n_
panarctic *D. pulex –* COX1	68	59	0.99	128	0.0248	0.0008
panarctic *D. pulex –* ND5	171	76	0.95	96	0.0396	0.0021
European *D. pulex*	3	3	1.00	8	0.0412	0.0018
European *D. pulicaria*	23	14	0.93	31	0.0306	0.0041
eastern *D. pulicaria*	10	9	0.98	12	0.0257	0.0015
western *D. pulicaria*	12	7	0.83	12	0.0179	0.0014
polar *D. pulicaria*	10	10	1.00	19	0.0227	0.0063
S. American *D. pulicaria* A	5	4	0.90	3	0.0000	0.0038
S. American *D. pulicaria* B	3	3	1.00	9	0.0281	0.0081
*D. middendorffiana*	6	6	1.00	12	0.0305	0.0033
*D. tenebrosa*	11	11	1.00	49	0.1055	0.0076

1 The number of individuals analyzed. Multiple individuals collected from the same location were removed.

2 The number of haplotypes.

3 The haplotype diversity, defined as the probability that two alleles randomly chosen from the sample are different.

4 The number of segregating sites.

5 π was estimated for nonsynonymous (π_n_) and synonymous (π_s_) sites using the correction of Jukes and Cantor.

In addition to panarctic *D. pulex* and the other eight lineages previously described by Colbourne et al. [12], two distinct lineages from South America are evident; South American *D. pulicaria* A (SA-A), and South American *D. pulicaria* B (SA-B) [13]. In our analysis, SA-B clusters within the pulicaria group, and panarctic *D. pulex* is the sister taxon to all the other pulicaria lineages (Figure 1). Mergeay et al. [13] identified a third South American lineage (SA-C), but it is not represented in our analysis as repeated attempts to amplify ND5 from individuals of this lineage have been unsuccessful (this study, Mergeay et al. [13]).

**Figure 2:**
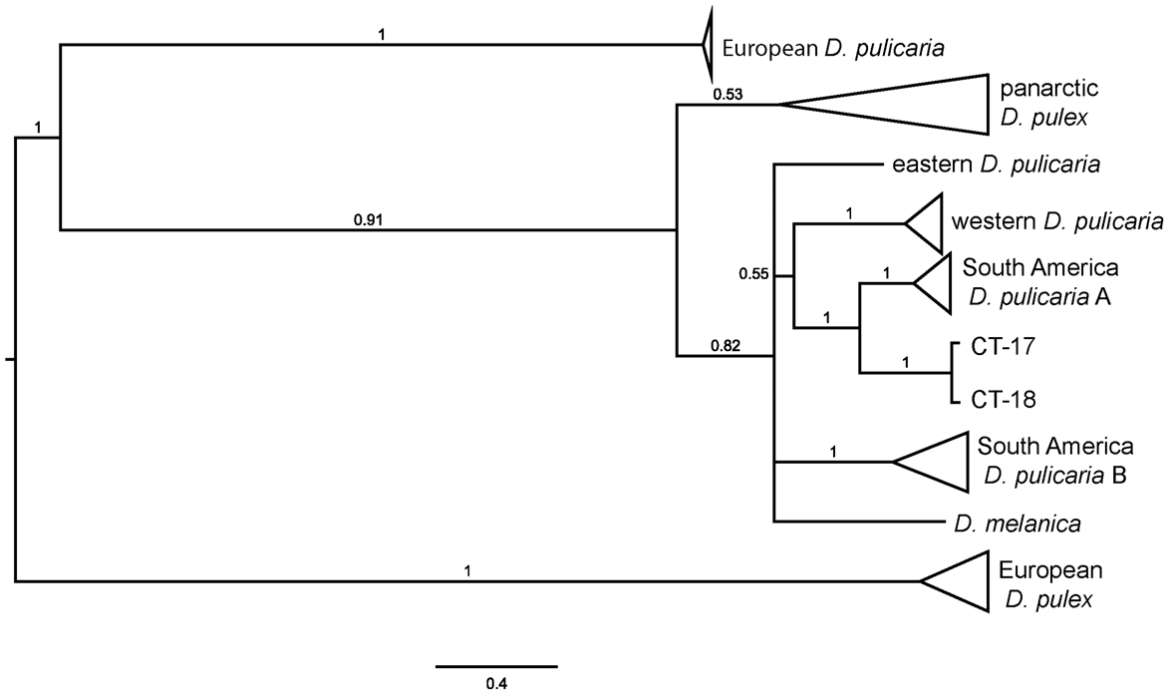
Consensus Bayesian phylogeny of the *Daphnia pulex* species complex **based on the mitochondrial ND5 and COX1 genes.** The alignment contains 98 sequences consisting of 496 nt of ND5 and 552 nt of COX1 with 295 polymorphic positions of which 258 are phylogenetically informative. The tree is rooted through the midpoint. Posterior probabilities are indicated on the nodes of the tree and are not shown if less than 0.80. Isolates CT-17 and CT-18 were collected from Connecticut, USA.

Bayesian phylogenetic analysis was also conducted on a dataset consisting of 98 sequences of ND5 combined with partial sequences of the mitochondrial *cytochrome* c *oxidase* subunit 1 (COX1) gene. This dataset includes 8 of the 12 lineages in the species complex, including SA-A and SA-B. After examining a variety of partitioning schemes, we obtained the lowest average standard deviation of split frequencies with unpartitioned data. The results from the different partitioning schemes are largely consistent; incongruence appeared only at branches with low posterior probability values. Both the ND5 (Figure 1) and the ND5+COX1 tree (Figure 2) suggest that SA-A and SA-B are derived from North American ancestors, and that there were at least two, and likely three introductions into South America from North America. For example, SA-A forms a strongly supported sister group to isolates CT-17 and CT-18 from Connecticut, USA.

**Figure 3:**
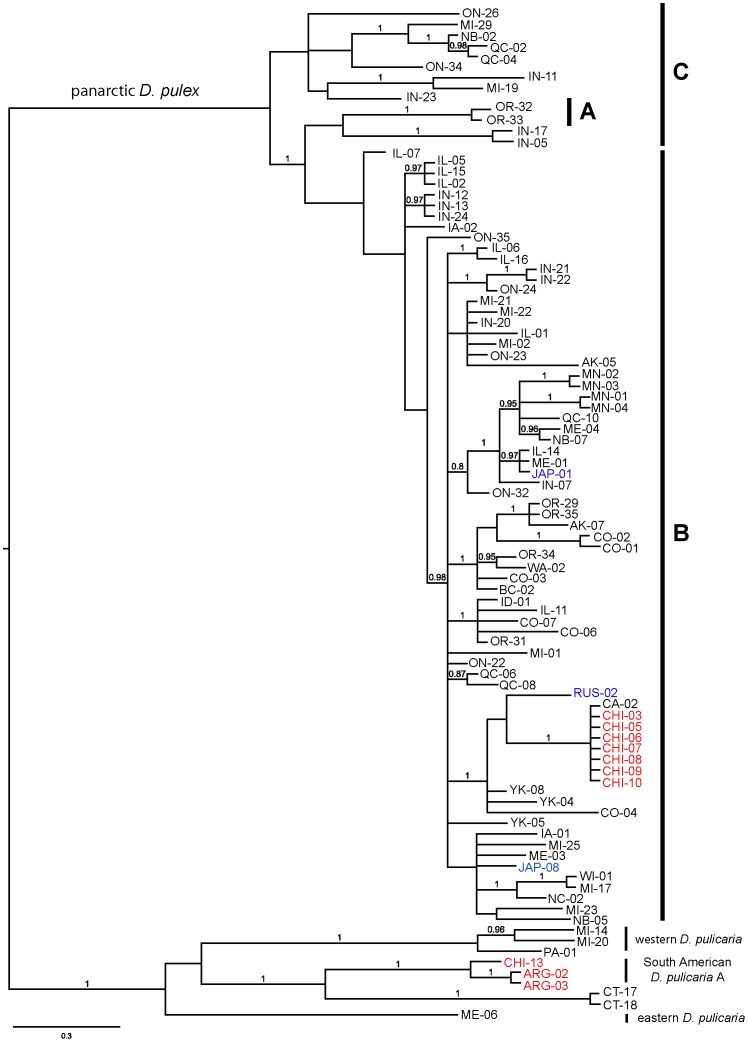
Consensus Bayesian phylogeny of the *Daphnia pulex* species complex based on the mitochondrial ND5 and COX1 genes. The alignment contains 95 sequences consisting of 762 nt of ND5 and 1386 nt of COX1 with 321 polymorphic positions of which 204 are phylogenetically informative. The tree is rooted through the midpoint. Posterior probabilities are indicated on the nodes of the tree and are not shown if less than 0.80. Taxon colors represent geographic locations as follows: black = North America, blue = east Asia, red = South America.

Analysis of 95 longer ND5+COX1 sequences representing four lineages (panarctic *D. pulex*, eastern and western *D. pulicaria*, SA-A, Figure 3), identified two of the three subgroups of panarctic *D. pulex* (A, B and C) identified by Paland et al. [14]. However, in contrast to their results, group A is not the sister taxon to the other two; instead, individuals from Paland et al.’s group C occupy this position. Moreover, their group C is paraphyletic to both group A and group B in our tree.

**Figure 4:**
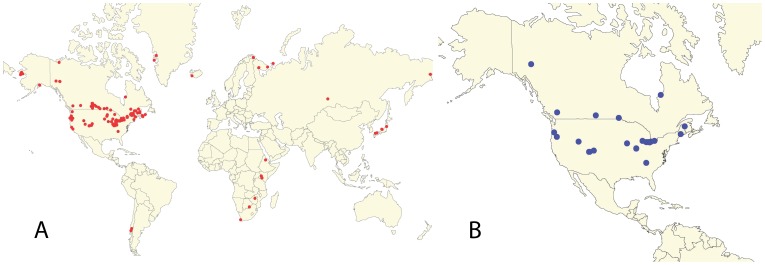
Location of collection sites for individuals in the panarctic *Daphnia pulex* lineage. (**a**) Red circles indicate collection sites for all individuals in the panarctic *D. pulex* lineage. (**b**) Blue circles indicate collection sites for individuals with the predicted ancestral haplotype for panarctic *D. pulex*. Sampling locations for all other individuals included in this study are provided in Figure S2.

Panarctic *D. pulex* is by far the most geographically widespread and well represented lineage in our analysis (65% of individuals analyzed), being observed on every continent except Australia and Antarctica (Figure 4, Table S1). Our new records from Japan, eastern Russia, western Alaska, and Chile provide large range extensions for this lineage. Even so, there is little obvious regional association of haplotypes in this lineage despite its large geographic range, with the exception of group A, which is restricted to western Oregon [14], [21]. Indeed, many of the individuals collected outside of North America share a very similar or even identical mitochondrial haplotype with individuals from North America (Figure 3, Figure S1). For example, individuals from two populations in Chile (Llanquihue Lake and Lake Riñuhue, Table S1) have the same panarctic *D. pulex* haplotype as individuals from California, USA (CA-02). In addition, haplotypes from Russia cluster with haplotypes from western North America (Colorado, California, Yukon) and those from Japan cluster with haplotypes from eastern (Maine) and midwestern USA (Iowa, Michigan).

**Figure 5:**
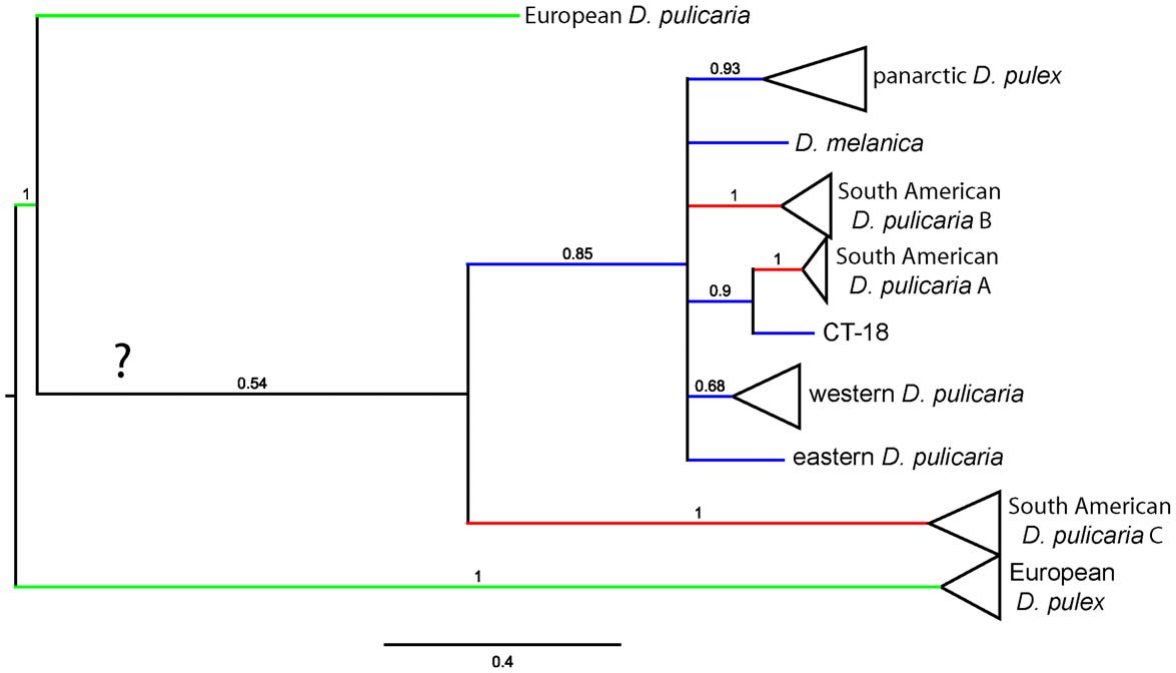
Consensus Bayesian phylogeny of the *Daphnia species* complex based on the mitochondrial COX1 gene. The alignment contains 52 sequences of length 552 nt with 151 polymorphic positions of which 112 are phylogenetically informative. Nine of the 12 lineages in the *Daphnia pulex* species complex, including all three South American lineages, are represented. The tree is rooted through the midpoint. Numbers at the nodes are Bayesian posterior probabilities and are not shown if less than 0.80. Branch colors correspond to continents as follows: green = Europe, blue = North America, red = South America. Individual CT-18 was collected from Connecticut, USA.

Because ND5 data are not available for SA-C, we constructed a tree from partial sequences of the COX1 gene from 52 individuals representing this lineage and seven of the other lineages. SA-C is the sister taxon to all the other pulicaria group lineages in this tree, as reported by Mergeay et al. [13], but relationships among these lineages are not well resolved with these data (Figure 5).

### Demographic History of Panarctic *D. pulex*

**Figure 6:**
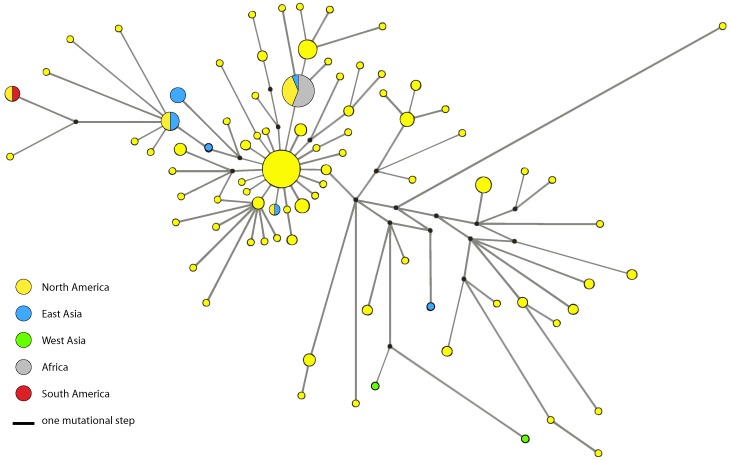
Median-joining haplotype network (ε = 0) for the panarctic *D. pulex* lineage. The network is based on the mitochondrial ND5 gene (496 nt, 173 individuals). Multiple individuals from the same location were removed. Each haplotype is represented by a circle whose area is proportional to the number of times the haplotype was observed. Median vectors, which represent either extant unsampled sequences or extinct ancestral sequences, are indicated by small black circles.

**Figure 7:**
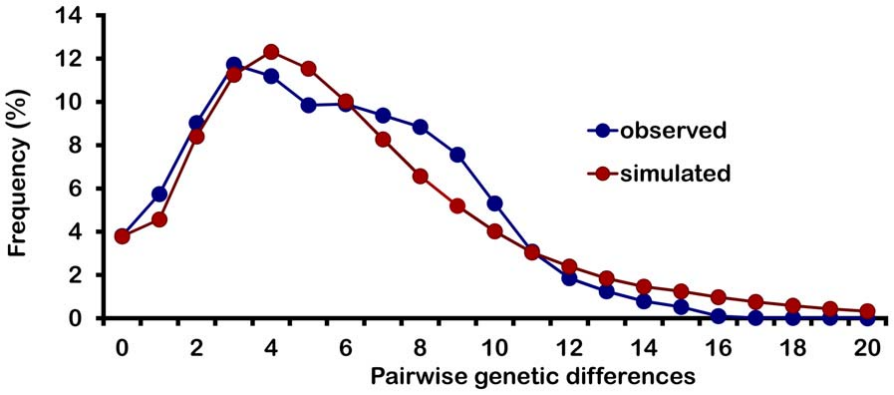
Mismatch distribution plot for the panarctic *D. pulex* lineage. This plot is based on the mitochondrial ND5 gene (496 nt, 173 individuals). Multiple individuals collected from the same location were removed.

Network analysis of 173 panarctic *D. pulex* using the ND5 alignment revealed 84 haplotypes. One haplotype was sampled from 27 sites throughout North America (Figure 4b) and is located in the center of the network (Figure 6), which shows the characteristic star-like pattern commonly associated with recent demographic or spatial expansion. A second widespread haplotype was observed in 16 sites from North America, Japan, and throughout Africa. A mismatch distribution plot based on this dataset is not significantly different from the expected distribution under an expansion model despite the appearance of a slightly jagged curve (P = 0.738, Figure 7). The raggedness index is low at 0.004 (P = 0.988) and values for Tajima’s *D* and Fu’s *Fs* (*D* = −2.212, *Fs* = −25.058, P<0.001) are significantly negative providing further support for recent population expansion. The τ parameter, which dates the initiation of the expansion in mutational time [22], was estimated as τ = 2.88 (95^th^ percentile confidence interval 1.45–7.38). Adopting Paland’s [23] direct estimate of the mitochondrial mutation rate in *D. pulex* (6.6×10^−8^), spatial expansion of panarctic *D. pulex* is estimated to have occurred 44,000 generations ago (95^th^ percentile confidence interval ranges from 22,000 to 113,000 generations ago).

## Discussion

### Phylogeny and Origin of the Pulicaria Group

Our phylogenetic analyses confirm the existence of at least 12 lineages in the *D. pulex* complex, and extend the geographic range of SA-A from one to two continents, and the range of panarctic *D. pulex* from three to five continents. SA-C is the sister taxon to the rest of the pulicaria group in our analysis (Figure 5), which is concordant with previous results [13], but we found that both SA-A and SA-B cluster within the pulicaria group, and that panarctic *D. pulex* is its sister taxon (Figure 2). In contrast, Mergeay et al. [13] found that both SA-B and SA-C cluster outside the rest of the pulicaria group, and consequently proposed that the pulicaria ancestor came from South America. All three SA lineages are as yet associated only with obligately parthenogenetic, polyploid individuals from high-altitude lakes and appear to be interspecific hybrids [13], [18], a situation that closely parallels that observed in arctic North America [16], [24]. Moreover, we found close relatives of SA-A in temperate North America (CT-17 and CT-18 from Connecticut), which suggests that both SA-A and SA-B are derived from a North American ancestor, consistent with the Holarctic origin proposed for this group [9], [17], [19]. Thus, SA-C is currently the only lineage in the pulicaria group without a close North American relative.

The Panama land bridge formed during the Pliocene and facilitated the movement, in both directions, of fauna between North and South America [25]. Successive expansion of *Daphnia* through Central and into South America (which were cooler during the Pliocene) could have occurred during this period, with subsequent extinction of the intervening lineages in regions that are now tropical. Indeed, Adamowicz et al. [8] noted a propensity for dispersal between these two continents throughout the genus, *Daphnia*. Discovery of the haplotypes from Connecticut (CT), and of panarctic *D. pulex* haplotypes in Chile that are identical to haplotypes in western USA, provide support for the hypothesis that members of the pulicaria group have moved from North to South America several times. Thus, the possibility remains that SA-C also exists but is yet undetected in North America, or has gone extinct there.

Adamowicz et al. [8] mapped continental regions onto the phylogeny of the entire genus *Daphnia* in order to explore the frequency and orientation of intercontinental speciation. Based on their analysis, it was not possible to determine the region from which the ancestor of the pulicaria group came (Figure 5). However, based on the Holarctic distribution of the *D. pulex* species complex, it seems unlikely that it came from South America. A more parsimonious hypothesis is that it colonized the Nearctic from Asia and spread to South America soon thereafter. Testing hypotheses about the origin of the pulicaria group will require even more extensive sampling in the temperate regions of South America, as well as the temperate and high-altitude regions of Central America.

### Demographic History of Panarctic *D. pulex*

Despite a near worldwide sampling effort and our discovery of panarctic *D. pulex* from Alaska, Central Asia, Japan, and South America, the only evidence of regionalism in this lineage is the A subgroup, which is restricted to Oregon, USA and is thought to have diverged during the Pleistocene in a refugium located south of the Cordilleran ice sheet [14], [21]. This lineage, to which the isolate whose full genome was sequenced [26] belongs, is also divergent at nuclear loci [21], [27], [28]. Otherwise, panarctic *D. pulex* has apparently undergone a Late Quaternary expansion and recently emigrated from North America to other continents. Even so, this lineage is primarily restricted to temperate regions (Figure 4) while other members of the *D. pulex* complex (*D. pulicaria*, *D. middendorffiana*, *D. tenebrosa*) dominate in the Arctic [15], and other species in the subgenus *Daphnia* dominate in the desert and subtropical regions of North America [29].

Panarctic *D. pulex* is by far the most prevalent lineage in our samples. Given that it has been studied more rigorously than other lineages in North America, and we incorporated a substantial amount of previously published data, its overrepresentation in our dataset may be partly due to sampling bias. Nevertheless, it is clear that panarctic *D. pulex* has a more extensive geographical range than any other lineage in the *D. pulex* complex with individuals sampled from five continents.

Past bottlenecks and subsequent range expansions are commonly documented for taxa that currently inhabit geographic regions that were glaciated during the Pleistocene [30]. Genetic signatures of such expansion include star-like haplotype networks with a common, presumably ancestral haplotype being central to other rarer haplotypes [31], [32], and unimodal plots of the number of differences between pairs of haplotypes (mismatch distribution, [22], [32]). In addition, significantly negative values of Tajima’s *D* and Fu’s *Fs* indicate an excess of low frequency variants, which can be attributed to expanding population size or range. Our results suggest that the panarctic *D. pulex* lineage is likely to have experienced spatial expansion in North America approximately 44,000 (22,000 to 113,000) generations ago based on the mitochondrial haplotype network (Figure 6), mismatch distribution (Figure 7), Tajima’s *D* and Fu’s *Fs* analyses. Assuming that Daphnia undergo between two and five generations per year depending on location, this would suggest that expansion occurred between 8,800 and 22,000 years ago. Although this estimate is more recent than others reported for this group [14], [20], it is not unreasonable given that the maximum extent of the last glacial advance in North America occurred about 21,000 years ago and ended about 10,000 years ago [33]. Moreover, it overlaps with the Hypsithermal or Holocene thermal maximum period of warming that occurred from about 9,000 to 4,000 years ago [34], and during which range expansion of other temperate flora and fauna is thought to have occurred [35].

There are some caveats to our finding of recent range expansion as other scenarios, such as ancient range expansion with high migration between neighboring demes [36], [37], can also mimic the signature of recent expansion. However, this scenario seems unlikely in the case of panarctic *D. pulex* as most of its range in North America was glaciated during the last glacial maximum. In addition, regional studies of genetic variation indicate that gene flow among populations on a local scale is typically very low [38], [39], [40], [41], although some obligately parthenogenetic clones have spread over very large geographic areas [9], [16]. Heterogeneity of mutation rates among sites [42], [43], and/or selective sweeps [32], [44] can also mimic the signature of population expansion, and we cannot exclude the possibility that one or both of these factors has contributed to the distribution of mitochondrial variation that we observed.

Morphological similarity across wide geographic areas coupled with high potential for long-distance dispersal via diapausing eggs originally led to the conclusion that many *Daphnia* species are cosmopolitan [17], [45]. However, subsequent morphological [17], [46] and genetic analyses showed that regionalism is more common [8], [15], [21], [47], [48], [49] and that many species are restricted to a single continent. Even so, cases of transcontinental establishment in cladocerans have been documented [8], [50], [51], [52], [53]. For example, eastern *D. pulicaria*, which was thought to occur only in North America, has now been reported in the High Tatra and Pyrenees mountains in Europe [54]. Moreover, we found that the clone currently spreading in Africa [9] has a haplotype that is widespread in North America (Figure 6, Figure S1), that a haplotype from Chile is identical to one from California, USA, and that haplotypes from Japan are very similar (but not identical) to the putative ancestral haplotype, suggesting that panarctic *D. pulex* has repeatedly traversed great distances to colonize new regions.

It has long been suggested that migratory birds act as long-distance dispersal vectors of invertebrate resting eggs [55], and a recent study has shown that the genetic structure of *Daphnia ambigua* and *Daphnia laevis* in North America is consistent with the movement of water fowl [56]. However, recent intercontinental dispersal of cladocerans has also been attributed to human activity [55] and indeed, Mergeay et al. [9] suggested that the panarctic *D. pulex* clone in Africa was introduced into Lake Naivasha (Kenya) from the USA during fish stocking programs in the late 1920s. However, they also suggested that dispersal by migrant waterfowl has helped to spread the clone throughout the continent.

There are four major migratory bird flyways between North and South America (Atlantic, Central, Mississippian and Pacific), and the occurrence of the same panarctic *D. pulex* haplotype in California and Chile is consistent with movement of resting eggs along the Pacific flyway. In addition, Weider et al. [15] suggested that panarctic *D. pulex* could have invaded northern Europe via waterfowl that migrate between wintering grounds in Europe and summer breeding grounds in arctic Canada, which could also explain the occurrence of eastern *D. pulicaria* in Europe [54]. Waterfowl are also known to migrate between eastern Asia and Alaska [57], which could account for the presence of panarctic *D. pulex* in Japan and Russia.

One hypothesis to explain the success of the ubiquitous panarctic *D. pulex* lineage outside its typical range in temperate North America is its frequent hybridization with other members of the pulicaria group, and the maintenance of these hybrid genotypes via obligate parthenogenesis. For example, no sexual populations of the pulicaria group have been detected in South America, and all the clones appear to be of hybrid origin [13], [18]. Moreover, when pulicaria-group populations are detected in desert or subtropical regions of North America they are typically *D. pulex* × *D. pulicaria* hybrids that reproduce by obligate parthenogenesis [29], as is the North American clone that is invading Africa [9]. Previous work has suggested that the lack of gene flow between regional populations of *Daphnia* is likely the result of priority effects and local adaptation, which makes it difficult for immigrant genotypes to integrate into well-established *Daphnia* communities [58]. In contrast, Mergeay et al. [9] highlighted the fact that the clone introduced to Africa was able to displace a genetically diverse, sexually reproducing indigenous species that was supposedly well-adapted to its environment in only 75 years. Further study of such *Daphnia* clones should provide important insights into the characteristics that make them such successful invaders of established *Daphnia* communities.

### Conclusions

Our phylogenetic analyses support the evolutionary relationships among eleven of the major mitochondrial lineages of the *D. pulex* species complex. However, we find little support for the hypothesis that the pulicaria group originated in South America. While most lineages within the species complex are restricted to one or two continents, the panarctic *D. pulex* lineage was detected on every continent except Australia and Antarctica. Despite this wide geographic range, no strong regionalism was detected, a pattern that differs from that observed in other cladocerans. In addition, our results suggest that this lineage has undergone recent expansion with some haplotypes spreading to more than one continent. The hypothesis that hybridization and maintenance of the resulting genotypes by obligate parthenogenesis has contributed to the recent and unusual geographic success of the panarctic *D. pulex* lineage warrants further study.

## Materials and Methods

### Taxonomic Sampling

We sought to examine individuals of the *D. pulex* complex from regions that had not been sampled extensively in the past, i.e., western Alaska, Japan, Russia, the Canadian prairies, South America, and the Southwestern United States. Species delimitation and nomenclature within this complex, and especially the pulicaria group, is problematic (reviewed in Mergeay et al. [13]) and we used the nomenclature based on phylogenetic analysis of mtDNA described by Colbourne et al. [12] and Mergeay et al. [13] for our samples.

We generated new sequences of 762 nt of the ND5 gene from 195 individuals and combined these with 203 published sequences [9], [12], [13], [14], [18], [54], [59], [60] to generate an ND5 dataset of 398 individuals representing all but one lineage in this species complex (Table S1). Sequences from previous studies with a substantial amount of missing nucleotide data (greater than 30 Ns in a single read) were not included in our analyses. We used *Daphnia* cf. *obtusa* from North America as an outgroup in the phylogenetic analysis of this dataset as it has been identified as a close relative of the *D.* *pulex* species complex with independent evidence [61].

We also amplified the COX1 gene from 66 of our DNA samples (mainly panarctic *D. pulex*), and added them to previously published sequences [59], [60] for a total of 95 COX1 sequences of 1386 nt. We added these sequences to our longer ND5 sequences (762 nt) to create a dataset representing four lineages with an alignment length of 2148 nt. We also generated a larger dataset representing eight lineages by combining 496-nt sequences of ND5 with 552-nt sequences of COX1 from our new and previously published sequences. New sequence data have been deposited in GenBank under accession numbers JX532724 - JX532790 (1386 nt COX1), JX532791 - JX532857 (762 nt ND5) and JX532858 - JX532985 (496 nt ND5).

### DNA Extraction, PCR Amplification, and Sequencing

Total genomic DNA was extracted from *Daphnia* using QuickExtract (Epicentre). Samples were homogenized in 50 µL of QuickExtract solution, incubated at 65°C for four hours, and 98°C for two min. PCR reactions were 50 µl total volume and contained 35 µl water, 5 µl 10X PCR buffer (1.5 mM MgCl_2_), 10 nmoles dNTPs, 15 pmoles of each primer, 1 U of *Taq* polymerase, and 25–50 ng of DNA template. PCR was conducted on an MJ Thermocycler with the following conditions: 40 cycles of 30 sec at 94°C, 30 sec at 48°C, and one min at 72°C; followed by one cycle of six min at 72°C. PCR amplicons were sequenced in both directions by High-Throughput Sequencing Solutions at the University of Washington, Department of Genome Sciences (Seattle, WA).

The primers ND5pulexF (5′GGGGTGTATCTATTAATTCG) and ND5pulexR (5′ATAAAACTCCAATCAACCTTG) were used to generate an amplicon of 897 nt from the ND5 gene. These primers were used for both PCR and sequencing reactions. The primers COX1pulexF (5′CCTACTCCTCGGCCATTTG) and COX1pulexR (5′GGGGATGCTCTATTTTGGAA) were used to generate an amplicon of 1679 nt containing the complete COX 1 gene. Internal sequencing primers were used for this locus and are as follows: COX1seqintR (5′TGAATCTTTAACCAACGGG) and COX1seqint_B (5′ CGTGAAGTGTGCCAAGTCAT).

### Phylogenetic Analyses, Sequence Divergence, and Diversity

DNA sequence electropherograms were edited and assembled with Sequencher 4.7 (Gene Codes Corp.) and CodonCode Aligner, and aligned with ClustalW in MEGA 4 [62]. The alignment for these protein coding regions was unambiguous and no indels were observed. We constructed phylogenetic trees using Bayesian inference in MrBayes 3.1.2 [63]. The effects of data partitioning were explored by conducting an analysis without partitioning, an analysis with the data partitioned by locus, and an analysis with the data partitioned by one noncoding and three codon positions (1^st^, 2^nd^, and 3^rd^ codon positions). Model selection for each partition was based on the Akaike information criterion in the program Modeltest 3.7 [64], [65]. The best-fitting model for each partition was employed in subsequent phylogenetic analysis. Default prior settings were used with the exception of the ‘ratepr’ parameter, which was set to ‘variable’ so that partitions could evolve at different rates. Branch lengths and topology were shared among partitions, but the substitution rate matrix, state frequency, and shape parameter of the gamma distribution were unlinked to allow separate parameter estimates.

Two independent and simultaneous Markov chain Monte Carlo (MCMC) analyses of fifteen heated and one cold chain were run for six million generations with sampling from the chain every 100 generations. After verifying chain convergence (average standard deviation of split frequencies <0.01), we discarded the initial 25% of trees as ‘burn-in’. A 50% majority-rule consensus tree with posterior probability (PP) values for each node was constructed from the remaining Bayesian trees. Branch support was the proportion of trees that contained a lineage, which represents the posterior probability of lineage existence, given the data and the model of evolution. Each phylogenetic analysis was repeated at least twice and the results were inspected with Tracer 1.4 [66].

Nucleotide diversity (π), defined as the average number of pairwise differences among DNA sequences [67], was estimated for each lineage and for the following categories of nucleotide sites in the ND5 gene: synonymous (π_s_), nonsynonymous (π_n_), and total (π_T_) using DNASP [68].

Genealogical relationships in panarctic *D. pulex* were further examined with median-joining haplotype networks using the program Network 4.5 [69]. The ε parameter was set to 0. We used the MP post-processing option, which removes all superfluous median vectors and links that are not contained in the shortest trees of the network. For network and subsequent demographic analyses, the panarctic *D. pulex* dataset was pruned so that one randomly chosen individual per collection site was included (Table S1). This pruning minimizes bias due to uneven sampling across geographic sites.

### Demographic History of Panarctic *D. pulex*

To elucidate the demographic history of panarctic *D. pulex*, frequency (mismatch) distributions of the number of differences between pairs of haplotypes [29], [33], [70], [71] were constructed with Arlequin 3.11 [72]. In addition, the raggedness index [73] was used to measure the smoothness of the pairwise mismatch plots. The simulated model of spatial expansion was compared to the observed mismatch distribution and the sum of square deviations (SSD) between the observed and expected distributions was tested with 10,000 permutation replicates. *P-*values were obtained by calculating the proportion of simulations that had an SSD that was equal to or larger than the observed SSD. A generalized non-linear least-square approach was employed in Arlequin to estimate the τ parameter, which is time to expansion in mutational time (τ = 2µt), where µ is the mutation rate for the entire haplotype and t is time in generations. Confidence intervals for τ were obtained by parametric bootstrapping [71].

We used Arlequin 3.11 [72] to calculate Tajima’s *D* and Fu’s *F_S_*, which are commonly used to detect departures from the neutral model, from panarctic *D. pulex* sequences. The significance of Tajima’s *D* was determined by generating random samples under the hypothesis of selective neutrality and population equilibrium using coalescent simulations. The significance of Fu’s *Fs* was determined by generating random samples under the hypothesis of neutrality and then estimating *P*-values as the proportion of *Fs* statistics that were less than or equal to the observed value [72].

## Supporting Information

Figure S1 **Full Bayesian phylogeny of the** ***Daphnia pulex*** **species complex from** **Figure 1** **.** This is a PDF file. The tree is based on 496 nt of the mitochondrial ND5 gene and shows all 398 individuals included in this study, plus the outgroup. Taxa represented on branches followed by a letter are given below. **A** CZE-02, CZE-04, CZE-06, GER-02, SWI-01, wSIB-10 **B** SAF-01, BOT-01, BOT-02, BOT-03, ETH-01, ETH-02, JAP-01, KEN-01, KEN-02, KEN-03, ZIM-01, AB-01, IL-13, IL-14, ME-01, ME-02, OR-06, SK-07, SK-10, SK-13 **C** ME-04, MN-01, MN-04, NB-06, NB-07, NY-01, QC-10, QC-17, QC-18 **D** BC-01, BC-02, CO-01, CO-02, CO-03, IA-01, ID-01, IL-10, IL-12, IN-20, MI-03, MI-12, MI-13, MI-21, MI-22, NB-03, NC-01, NC-02, NU-01, NU-03, ON-07, ON-08, ON-10, ON-11, ON-17, ON-21, ON-22, ON-28, ON-29, ON-32, OR-28, OR-29, OR-30, OR-31, SK-05, YK-05, YK-06 **E** RUS-01, RUS-02, SK-04, wSIB-03, YK-01, YK-02, YK-07, YK-08 **F** CA-01, CA-02, CA-03, CHI-01, CHI-02, CHI-03, CHI-04, CHI-05, CHI-06, CHI-07, CHI-08, CHI-09, CHI-10 **G** JAP-02, JAP-04, JAP-05, JAP-06, JAP-07, JAP-10, JAP-11 **H** OR-03, OR-07, OR-08, OR-11, OR-18, OR-19 **I** NB-01, NB-02, NS-03, NS-04, QC-02, QC-04 **J** CHI-12, CHI-14, CHI-15, ARG-01, ARG-02, ARG-03, ARG-09.
(PDF)

Figure S2 **Collection sites for lineages of the** ***Daphnia pulex*** **species complex included in this study, excluding panarctic** ***D. pulex*** **.** This is a PDF file. Colors are used to indicate lineages as follows: black = European *D. pulex* orange = European *D. pulicaria* green = eastern *D. pulicaria* magenta = western *D. pulicaria* light blue = polar *D. pulicaria* brown = *D. middendorffiana* dark purple = *D. tenebrosa* light purple = S. American *D. pulicaria* A pink = S. American *D. pulicaria* B.
(PDF)

Table S1 **Individuals of the** ***Daphnia pulex*** **species complex included in this study.** This Excel spreadsheet provides information on all individuals included in this study. The individuals that were included in each phylogenetic or network analysis are indicated in the last 5 columns. Latitude and longitude of collection sites for some previously published accessions were estimated with Google Earth.
(XLS)
