# Supplementary material for: Transcontinental Phylogeography of the Daphnia pulex Species Complex
Source: PLoS One. 2012 Oct 3;7(10):e46620. doi: 10.1371/journal.pone.0046620 (PMC3463573; doi:10.1371/journal.pone.0046620)
Supplement: Figure S1 — Full Bayesian phylogeny of the Daphnia pulex species complex from Figure 1 . This is a PDF file. The tree is based on 496 nt of the mitochondrial ND5 gene and shows all 398 individuals included in this study, plus the outgroup. Taxa represented on branches followed by a letter are given below. A CZE-02, CZE-04, CZE-06, GER-02, SWI-01, wSIB-10 B SAF-01, BOT-01, BOT-02, BOT-03, ETH-01, ETH-02, JAP-01, KEN-01, KEN-02, KEN-03, ZIM-01, AB-01, IL-13, IL-14, ME-01, ME-02, OR-06, SK-07, SK-10, SK-13 C ME-04, MN-01, MN-04, NB-06, NB-07, NY-01, QC-10, QC-17, QC-18 D BC-01, BC-02, CO-01, CO-02, CO-03, IA-01, ID-01, IL-10, IL-12, IN-20, MI-03, MI-12, MI-13, MI-21, MI-22, NB-03, NC-01, NC-02, NU-01, NU-03, ON-07, ON-08, ON-10, ON-11, ON-17, ON-21, ON-22, ON-28, ON-29, ON-32, OR-28, OR-29, OR-30, OR-31, SK-05, YK-05, YK-06 E RUS-01, RUS-02, SK-04, wSIB-03, YK-01, YK-02, YK-07, YK-08 F CA-01, CA-02, CA-03, CHI-01, CHI-02, CHI-03, CHI-04, CHI-05, CHI-06, CHI-07, CHI-08, CHI-09, CHI-10 G JAP-02, JAP-04, JAP-05, JAP-06, JAP-07, JAP-10, JAP-11 H OR-03, OR-07, OR-08, OR-11, OR-18, OR-19 I NB-01, NB-02, NS-03, NS-04, QC-02, QC-04 J CHI-12, CHI-14, CHI-15, ARG-01, ARG-02, ARG-03, ARG-09. (PDF) [file pone.0046620.s001.pdf]

Figure S1. Full Bayesian phylogeny of the *Daphnia pulex* species complex from Figure 1.

The tree is based on 496 nt of the mitochondrial ND gene and shows all 398 individuals included in this study, plus the outgroup. Taxa represented on branches followed by a letter are given below.

A CZE-02, CZE-04, CZE-06, GER-02, SWI-01, wSIB-10

B SAF-01, BOT-01, BOT-02, BOT-03, ETH-01, ETH-02, JAP-01, KEN-01, KEN-02, KEN-03, ZIM-01; AB-01, IL-13, IL-14, ME-01, ME-02, OR-06, SK-07, SK-10, SK-13

C ME-04, MN-01, MN-04, NB-06, NB-07, NY-01, QC-10, QC-17, QC-18

D BC-01, BC-02, CO-01, CO-02, CO-03, IA-01, ID-01, IL-10, IL-12, IN-20, MI-03, MI-12, MI-13, MI-21, MI-22, NB-03, NC-01, NC-02, NU-01, NU-03, ON-07, ON-08, ON-10, ON-11, ON-17, ON-21, ON-22, ON-28, ON-29, ON-32, OR-28, OR-29, OR-30, OR-31, SK-05, YK-05, YK-06

E RUS-01, RUS-02, SK-04, wSIB-03, YK-01, YK-02, YK-07, YK-08

F CA-01, CA-02, CA-03, CHI-01, CHI-02, CHI-03, CHI-04, CHI-05, CHI-06, CHI-07, CHI-08, CHI-09, CHI-10

G JAP-02, JAP-04, JAP-05, JAP-06, JAP-07, JAP-10, JAP-11

H OR-03, OR-07, OR-08, OR-11, OR-18, OR-19

I NB-01, NB-02, NS-03, NS-04, QC-02, QC-04

J CHI-12, CHI-14, CHI-15, ARG-01, ARG-02, ARG-03, ARG-09

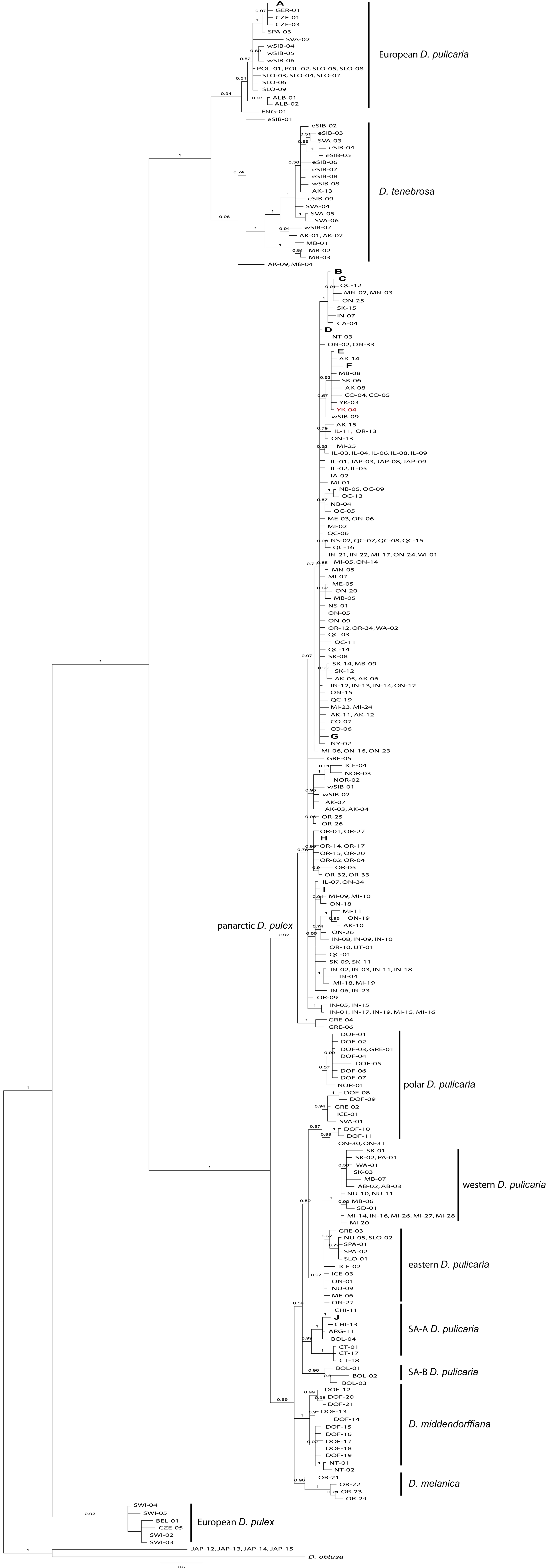

A
GER-01
CZE-01
CZE-03
SPA-03
SVA-02
wSIB-04
wSIB-05
wSIB-06
POL-01, POL-02, SLO-05, SLO-08
SLO-03, SLO-04, SLO-07
SLO-06
SLO-09
ALB-01
ALB-02
ENG-01
European D. pulicaria
eSIB-01
eSIB-02
eSIB-03
SVA-03
eSIB-04
eSIB-05
eSIB-06
eSIB-07
eSIB-08
wSIB-08
AK-13
eSIB-09
SVA-04
SVA-05
SVA-06
wSIB-07
AK-01, AK-02
MB-01
MB-02
MB-03
AK-09, MB-04
D. tenebrosa
B
C
QC-12
MN-02, MN-03
ON-25
SK-15
IN-07
CA-04
D
NT-03
ON-02, ON-33
E
AK-14
F
MB-08
SK-06
AK-08
CO-04, CO-05
YK-03
YK-04
wSIB-09
AK-15
IL-11, OR-13
ON-13
MI-25
IL-03, IL-04, IL-06, IL-08, IL-09
IL-01, JAP-03, JAP-08, JAP-09
IL-02, IL-05
IA-02
MI-01
NB-05, QC-09
QC-13
NB-04
QC-05
ME-03, ON-06
MI-02
QC-06
NS-02, QC-07, QC-08, QC-15
QC-16
IN-21, IN-22, MI-17, ON-24, WI-01
MI-05, ON-14
MN-05
MI-07
ME-05
ON-20
MB-05
NS-01
ON-05
ON-09
OR-12, OR-34, WA-02
QC-03
QC-11
QC-14
SK-08
SK-14, MB-09
SK-12
AK-05, AK-06
IN-12, IN-13, IN-14, ON-12
ON-15
QC-19
MI-23, MI-24
AK-11, AK-12
CO-07
CO-06
G
NY-02
MI-06, ON-16, ON-23
GRE-05
ICE-04
NOR-03
NOR-02
wSIB-01
wSIB-02
AK-07
AK-03, AK-04
OR-25
OR-26
OR-01, OR-27
H
OR-14, OR-17
OR-15, OR-20
OR-02, OR-04
OR-05
OR-32, OR-33
IL-07, ON-34
I
MI-09, MI-10
ON-18
MI-11
ON-19
AK-10
ON-26
IN-08, IN-09, IN-10
OR-10, UT-01
QC-01
SK-09, SK-11
IN-02, IN-03, IN-11, IN-18
IN-04
MI-18, MI-19
IN-06, IN-23
OR-09
IN-05, IN-15
IN-01, IN-17, IN-19, MI-15, MI-16
GRE-04
GRE-06
panarctic D. pulex
DOF-01
DOF-02
DOF-03, GRE-01
DOF-04
DOF-05
DOF-06
DOF-07
NOR-01
DOF-08
DOF-09
GRE-02
ICE-01
SVA-01
DOF-10
DOF-11
ON-30, ON-31
polar D. pulicaria
SK-01
SK-02, PA-01
WA-01
SK-03
MB-07
AB-02, AB-03
NU-10, NU-11
MB-06
SD-01
MI-14, IN-16, MI-26, MI-27, MI-28
MI-20
western D. pulicaria
GRE-03
NU-05, SLO-02
SPA-01
SPA-02
SLO-01
ICE-02
ICE-03
ON-01
NU-09
ME-06
ON-27
eastern D. pulicaria
CHI-11
J
CHI-13
ARG-11
BOL-04
CT-01
CT-17
CT-18
SA-A D. pulicaria
BOL-01
BOL-02
BOL-03
SA-B D. pulicaria
DOF-12
DOF-20
DOF-21
DOF-13
DOF-14
DOF-15
DOF-16
DOF-17
DOF-18
DOF-19
NT-01
NT-02
D. middendorffiana
OR-21
OR-22
OR-23
OR-24
D. melanica
SWI-04
SWI-05
BEL-01
CZE-05
SWI-02
SWI-03
European D. pulex
JAP-12, JAP-13, JAP-14, JAP-15
D. obtusa
0.5
