# Supplementary material for: Transcontinental Phylogeography of the Daphnia pulex Species Complex
Source: PLoS One. 2012 Oct 3;7(10):e46620. doi: 10.1371/journal.pone.0046620 (PMC3463573; doi:10.1371/journal.pone.0046620)

**Figure S2. Collection sites for lineages of the *Daphnia pulex* species complex included in this study, excluding panarctic *D. pulex*.**

Colors are used to indicate lineages as follows:

black = European *D. pulex*

orange = European *D. pulicaria*

green = eastern *D. pulicaria*

magenta = western *D. pulicaria*

light blue = polar *D. pulicaria*

brown = *D. middendorffiana*

dark purple = *D. tenebrosa*

light purple = S. American *D. pulicaria* A

pink = S. American *D. pulicaria* B

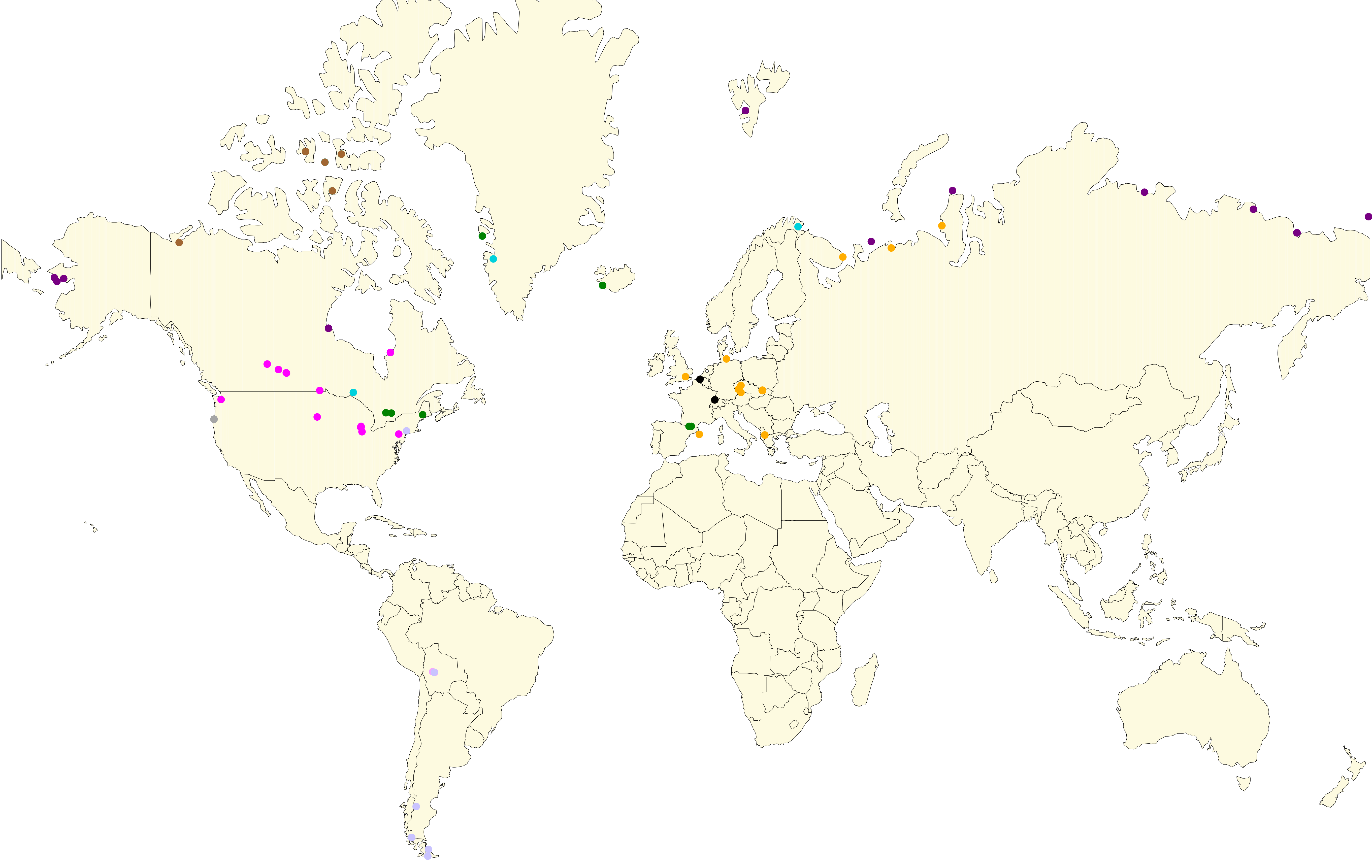

Supplement: Figure S2 — Collection sites for lineages of the Daphnia pulex species complex included in this study, excluding panarctic D. pulex . This is a PDF file. Colors are used to indicate lineages as follows: black = European D. pulex orange = European D. pulicaria green = eastern D. pulicaria magenta = western D. pulicaria light blue = polar D. pulicaria brown = D. middendorffiana dark purple = D. tenebrosa light purple = S. American D. pulicaria A pink = S. American D. pulicaria B. (PDF) [file pone.0046620.s002.pdf]
